# Reaction of Cysteine and Selenocysteine with a Model Au(I)-N-Heterocyclic Carbene Complex: A QM and QM/MM MD Comparative Study

**DOI:** 10.1021/acs.inorgchem.6c04543

**Published:** 2026-10-15

**Authors:** Milan Říha, Markéta Munzarová, Jaroslav V. Burda

**Affiliations:** † Department of Chemistry, Faculty of Natural Sciences, Masaryk University, Kamenice 5, Brno 62500, Czech Republic; ‡ Department of Chemical Physics and Optics, Faculty of Mathematics and Physics, 37740 Charles University, Ke Karlovu 3, Prague 2 121 16, Czech Republic

## Abstract

The substitution reaction of a model Au­(I)-N-heterocyclic carbene (NHC) complex with cysteine and selenocysteine represents a key elementary step in the inhibition mechanism of thioredoxin reductase by gold-based metallodrugs. We present a systematic quantum-mechanical (QM) and combined QM/MM investigation of this reaction with the aim of assessing the consistency and reliability of activation and reaction free energies obtained at different theoretical levels. The reaction was first studied at the QM level using several density functional theory (DFT) functionals and basis sets and subsequently within a QM/MM framework, employing multiple QM descriptions of the reactive region and explicit molecular-mechanics (MM) treatment of the solvent (water). Comparison of QM and QM/MM results reveals how the inclusion of the surrounding environment affects both activation barriers and reaction energies. Several protonation states of cysteine and selenocysteine were considered, and a pH-dependent model was constructed using a grand-canonical transformation of the free energy. Combining QM/MM reaction energetics with p*K*_a_ values yielded pH-dependent Gibbs–Alberty free energy profiles with a coordination-preference switch at pH ≈ 8. In acidic and neutral solutions the Au­(I) complex coordinates with selenocysteine, while in a basic environment, coordination with cysteine is energetically more stable.

## Graphical abstract

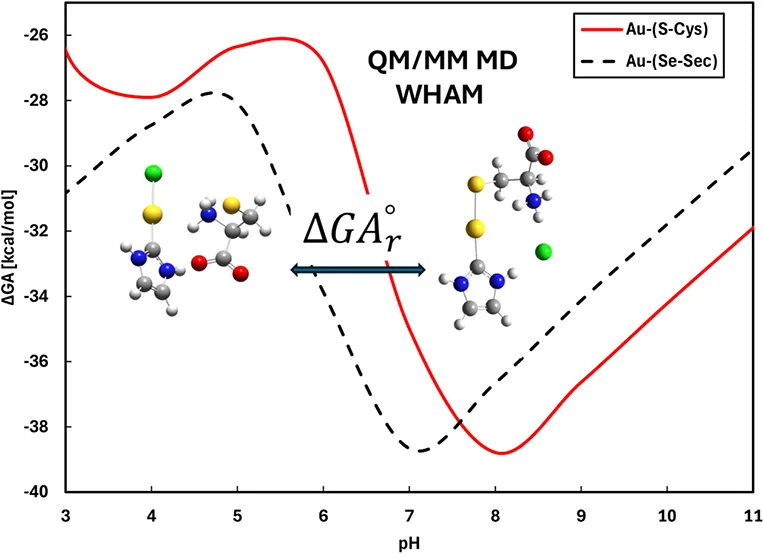

## Introduction

Reactive oxygen species (ROS) are continuously generated in aerobic cells as byproducts of mitochondrial oxidative metabolism, although additional cellular stressors can further stimulate their production.^1–3^ At low physiological levels, ROS function as important redox signaling molecules regulating cell proliferation and survival, and transcriptional programs.^1,2,4^ However, excessive ROS generation or an insufficient antioxidant defense leads to oxidative stress, resulting in oxidative damage and contributing to diverse pathological conditions including cancer and degenerative diseases.^1,4–6^

Oxidative stress promotes apoptosis and contributes to tumorigenesis through oxidative damage and disruption of redox signaling pathways.^1,5–7^ To maintain redox homeostasis, cells rely primarily on two major antioxidant systems: the glutathione system, which includes enzymes such as glutathione peroxidases (GPx), glutathione reductase (GR), and glutaredoxins (Grx), and the thioredoxin system.^8–10^ The thioredoxin system consists of the small protein—thioredoxin (Trx), enzyme—a thioredoxin reductase (TrxR), and cofactors—NADPH and FAD, forming one of the principal cellular redox defense networks responsible for preserving a reducing intracellular environment.^8,9,11^ Mammalian thioredoxin reductases are selenium-containing flavoproteins that require a C-terminal selenocysteine residue for catalytic activity.^8,12^ The presence of Sec in the active site confers enhanced nucleophilicity and distinct redox properties compared to analogous cysteine-containing enzymes, owing to the lower p*K*_a_ and higher chemical reactivity of Sec’s selenol group.^12^ Reduced thioredoxin influences numerous cellular processes, including DNA synthesis, regulation of transcription factors, and protection against oxidative stress.^13^ Importantly, the thioredoxin system is frequently upregulated in cancer cells, where elevated TrxR expression contributes to tumor growth, resistance to apoptosis, adaptation to chronic oxidative stress, and poorer patient prognosis.^14–16^

Metal-based drugs have long been investigated as anticancer agents following the discovery of cisplatin, which ultimately revolutionized chemotherapy.^17,18^ Inspired by platinum’s success, a variety of other metal complexes, including gold compounds, have been explored for cancer treatment.^14,19,20^ Au­(I) complexes were initially used clinically for rheumatoid arthritis, but subsequent studies revealed significant anticancer potential for these gold drugs.^21,22^ Gold compounds also exhibit a mechanism of action distinct from that of platinum drugs, as they generally show only weak interactions with DNA and instead preferentially coordinate to protein sites containing soft nucleophilic residues such as cysteine and selenocysteine (consistent with the soft Lewis acid character of Au­(I)).^14,23^ The strong affinity of Au­(I) for thiolate and selenolate groups consequently makes thiol- and selenol-containing enzymes key biological targets of gold-based agents.^22,24^

Auranofin, a clinically approved Au­(I) complex, has been shown to inhibit thioredoxin reductase by selectively targeting the mitochondrial TrxR isoform and induce oxidative stress-mediated apoptosis in cancer cells.^14,16,25–27^ Among modern gold-based drug candidates, complexes featuring N-heterocyclic carbene (NHC) ligands have attracted particular interest. NHC ligands are strong σ-donors that form robust Au–C bonds, thereby enhancing the stability of gold­(I) complexes in biological environments by resisting ligand substitution.^28–30^ Gold­(I)–NHC complexes often exhibit high cytotoxicity and potent inhibition of thioredoxin reductase thereby elevating intracellular ROS levels and promoting apoptotic cancer cell death.^28,30,31^

Recently, Au­(I) complexes with modified NHC were found to be more active against both colon and breast cancer cells in comparison with cisplatin or with auranofin.^32^ Particularly, the dinuclear Au­(I) phosphane complexes have significantly lower IC_50_ values against colorectal or breast cancer cells, as reported by Sulaiman et al.^33^ and Abogosh et al.^34^ New thiolate gold­(I) complexes that inhibit the proliferation of different human cancer cells, were reported by Abas et al.^35^ and showed higher cytotoxic activity than cisplatin. Strong antiproliferative effects of Au­(I) complexes with NHC ligands were also reported in another study involving lung, colon, and breast cancer cell lines.^36^ Despite extensive experimental investigations, the detailed mechanism, by which Au­(I)–NHC complexes react with cysteine- and selenocysteine-containing sites, remains incompletely understood. Computational chemistry has become an indispensable tool for elucidating the mechanism of action of metallodrugs.^37–39^ In particular, DFT calculations enable detailed interrogation of metal–ligand interactions, reaction pathways, and activation energy barriers for proposed mechanisms.^38,40^ Hybrid quantum mechanics/molecular mechanics (QM/MM) approaches combine an accurate quantum description of the reactive site with a classical representation of the protein and solvent environment.^41–44^

Recent quantum-mechanical studies have begun to probe model reactions of Au­(I)–NHC complexes with cysteine and selenocysteine and have highlighted the importance of protonation equilibria and pH effects on the reaction pathway.^45^ Moreover, theoretical models indicate that the substitution of a chlorido ligand of the Au­(I)-NHC complex by Cys/Sec is energetically favorable primarily when the nucleophilic Cys/Sec center is deprotonated. These findings underscore the critical role of the local chemical environment and protonation state.^46^ Recent studies have accordingly applied DFT and QM/MM methodologies to examine the reactivity of Au­(I)–NHC complexes in aqueous solution and within models of biological systems.^47^

In this context, a rigorous computational study of the substitution reactions between Au­(I)–NHC complexes, as a model of real gold NHC metallo-drugs, and Cys/Sec residues is undertaken to elucidate the molecular determinants of TrxR inhibition. A deeper mechanistic insight into how gold-based inhibitors inactivate TrxR is achieved by systematically comparing pure QM cluster models with QM/MM simulations and by evaluating the influence of protonation states and environmental factors. One of our main objectives is to perform a Legendre transformation, which allows to express free energy dependent on pH as an inner variable.^48,49^ Based on the Gibbs free energies and p*K*_a_ values for individual forms of Cys/Sec amino acids and their Au­(I)-coordinated variants, pH-dependent Gibbs–Alberty free energies were obtained using a grand-canonical transformation.

## Computational Details

### QM Methods

All quantum-chemical calculations were performed using several DFT functionals. Geometry optimizations were carried out for all reactants, products, and transition states using several widely employed functionals and basis sets. Specifically, the B3LYP,^50–52^ B3PW91,^50,51,53^ M06–2X,^54^ and PBE0 ^55^ functionals were employed. Based on the evaluation of the energy profiles of the considered reactions, the B3LYP and PBE0 functionals were chosen for further QM/MM calculations and construction of free energy profiles in dependence on pH of surrounding environment.

Also, several basis sets were compared to balance computational accuracy and efficiency.^56^ The most flexible basis set considered in this study was (i) the triple-ζ reference level (TZ), which employs 6–311++G­(2df,2pd) for H, C, N, and O atoms together with corresponding Stuttgart–Dresden relativistic effective core potentials for Au, Cl, and S/Se (ECP10MWB for chlorine and sulfur, ECP28MWB for selenium, ECP60MWB for gold^57^), with consistent extension of polarization and diffuse functions,^45^ (denoted as TZ hereafter). This was followed by (ii) the double-ζ 6–31++G­(d,p) for H, C, N, and O atoms combined with the Stuttgart–Dresden basis for Au, Cl, and S/Se also consistently extended by corresponding pseudoorbitals (labeled as DZ1). Finally, (iii) basis set 6–31G­(d,p) where diffuse functions were kept only on oxygen, together with diffuse-free Stuttgart–Dresden basis sets where polarization functions were applied to Au, Cl, and S/Se (labeled as DZ3). For additional computational efficiency and comparison purposes, the aforementioned functionals were also combined with the LANL2DZ basis set and pseudopotential^58^ for all atoms in selected calculations. Further details of the employed computational levels are provided in the Supporting Information.

Dispersion interactions were consistently included in all calculations using Grimme’s empirical dispersion correction with Becke–Johnson damping (GD3BJ).^59^ Solvent effects were modeled using the conductor-like polarizable continuum model (CPCM)^60^ employing the UFF cavities.^61^ Vibrational frequency analysis was performed at the same level of theory as the corresponding geometry optimizations to verify the nature of all stationary points and to obtain zero-point energy and thermal corrections. All Gibbs free energies reported in this work correspond to a temperature of 298.15 K. Standard statistical thermodynamics as implemented in quantum-chemical programs assumes an ideal-gas reference state within the rigid rotor and harmonic oscillator (RRHO) model and therefore tends to overestimate translational and rotational entropy contributions under condensed-phase conditions. To correct for this limitation, Wertz-type entropy reduction^62,63^ was applied to all computed Gibbs free energies. The corrected entropy was evaluated according to:

1 $$S=S_{gas}^{vib}+0.54\cdot \left(S_{gas}^{trans}+S_{gas}^{rot}-14.3\right)+8.0$$

where $S_{\mathrm{gas}}^{\mathrm{vib}}$ , $S_{\mathrm{gas}}^{\mathrm{trans}}$ and $S_{\mathrm{gas}}^{\mathrm{rot}}$ denote the vibrational, translational, and rotational entropy components, respectively, with −14.3 and 8.0 kcal.mol^–1^.K^–1^ reflecting the dilution and compression effects during the transition from an ideal gas phase to liquid solution.

Reaction rate constants were estimated using Eyring’s transition-state theory,^64^ in which the Wertz-corrected activation free energies Δ*G*≠were used:

2 $$k=\frac{k_{B}T}{h}\cdot e^{-\Delta G^{\neq }/RT}$$

at *T* = 298.15 K, *k*_*B*_ is the Boltzmann’s constant, and *h* is Planck’s constant.

### QM/MM MD Approach

In contrast to the purely QM approach employing implicit solvent models, the QM/MM methodology explicitly accounts for the solvent environment by including a large number of water molecules. While this explicit treatment provides a more realistic description of solvation effects, it also necessitates statistical averaging of the system’s energetics over an ensemble generated by molecular dynamics (MD) simulations. In the present QM/MM setup, the quantum mechanically treated region (QM region) comprised the gold complex associated with amino acid within G09 program suite. The remaining part of the system (water as the explicit solvent) was described at the molecular-mechanical level evaluated in Amber 14 software package. The QM/MM interface employed electrostatic embedding, allowing the electronic structure of the QM subsystem to respond to the electrostatic field generated by the point charges from the MM environment.

The molecular-mechanical parameters for the gold complex were derived using the General Amber Force Field (GAFF)^65^ in combination with RESP^66,67^ atomic charges obtained from fitted electrostatic potentials. Nonbonded and bonded parameters for the gold atom were adopted from our previous parametrization study.^68^ For the selenium atom, the parameters were adopted from Pedron et al.^69^ The MM environment consisted of a cubic simulation box containing approximately 8000 TIP3P^70^ water molecules, constructed using the *tleap* module of the Amber 14 ^71^ software. Prior to QM/MM MD propagation step, the system was subjected to energy minimization followed by equilibration in the NVT and NPT ensembles heated to 300.0 K using a Langevin thermostat^72^ and Berendsen barostat.^73^ All QM/MM molecular dynamics simulations were performed under 3d periodic boundary conditions (PBC). This setting QM/MM was used for determination of free energy surfaces of all explored systems.

The reaction coordinate *ξ* used for QM/MM free-energy determination was defined as

3 $$\xi =\frac{\left(R_{Au-Cl}-R_{Au-S/Se}\right)-\left(R_{Au-Cl}^{react}-R_{Au-S/Se}^{react}\right)}{\left(R_{Au-Cl}^{prod}-R_{Au-S/Se}^{prod}\right)-\left(R_{Au-Cl}^{react}-R_{Au-S/Se}^{react}\right)}$$

where *R*_*X*_ correspond to the instantaneous bond lengths, and $R_{X}^{react}$ and $R_{X}^{prod}$ represent the reference bond lengths for the Au–Cl and Au–S/Se bonds obtained from QM-optimized reactant and product supermolecules. This definition allows the reaction coordinate to be conveniently scaled to the interval *ξ* ∈ [0,1]. Within this interval, 14–17 equidistant umbrella sampling windows were generated. For each window, QM/MM MD simulations were performed with a time step of 0.5 fs and a total sampling time of 10 ps. In each window, the Au–Cl and Au–S/Se distances were restrained using harmonic bias potentials with a force constant of 100 kcal·mol^–1^ ·Å^–2^.

The QM region was treated using the B3LYP and PBE0 functionals together with basis sets described in the QM part of the Computational Details. The resulting umbrella sampling trajectories were analyzed using both the Weighted Histogram Analysis Method (WHAM)^74,75^ and the Umbrella Integration (UI)^76^ approach to construct the final free-energy profiles.

### Free Energy Distribution in QM/MM

In standard quantum-chemical calculations, reaction profiles are commonly obtained by evaluating energies of reactants, transition states, and products directly from optimized stationary points on the potential energy surface. In contrast, the construction of free-energy profiles within a QM/MM framework is substantially more demanding, as it requires explicit consideration of entropy contributions arising primarily from the solvent and the molecular environment, which cannot be captured by single-point energy evaluations.

Therefore, in QM/MM simulations, free energies must be extracted from statistical sampling along a chosen reaction coordinate. In the present work, umbrella sampling^77^ was employed to compute free energy profiles. The reaction coordinate *ξ* was partitioned into a series of overlapping windows, each sampled independently under the influence of a bias potential to ensure sufficient exploration of the configurational space along the entire reaction pathway. A harmonic bias potential in the form

4 $$w_{i}\left(\xi \right)=\frac{1}{2}K_{i}{\left(\xi -\xi_{i}^{0}\right)}^{2}$$

was applied in each window *i*, where *K*_*i*_ denotes the force constant and $\xi_{i}^{0}$ is the reference value of the reaction coordinate. From the biased probability distribution $P_{i}^{b}\left(\xi \right)$ , the free energy *A*_*i*_(*ξ*) within a given window was evaluated according to:

5 $$A_{i}\left(\xi \right)=-\frac{1}{\beta }\ln P_{i}^{b}\left(\xi \right)-w_{i}\left(\xi \right)+F_{i}$$

where *β* = (*k*_B_ *T*)^−1^ and *F*_*i*_ represents the free energy offset associated with the applied bias potential. This offset is given by

6 $$F_{i}=-\frac{1}{\beta }\ln\langle e^{-\beta w_{i}}\rangle$$

with angular brackets denoting ensemble averaging. To reconstruct the global free-energy profile along the full reaction coordinate, the WHAM was employed, which combines the biased probability distributions obtained from individual windows into a single unbiased distribution,

7 $$P^{u}\left(\xi \right)=\overset{N_{w}}{\underset{i=1}{\sum }}p_{i}\left(\xi \right)P_{i}^{b}\left(\xi \right)$$

where the weights *p*_*i*_(*ξ*) are determined self-consistently by minimizing statistical errors and depend on the free-energy shifts *F*_*i*_. eqs 5 and 6 are solved iteratively until convergence is achieved.

Because harmonic bias potentials were used throughout, the free-energy profiles were additionally analyzed using UI approach, which provides an analytical alternative based on direct evaluation of mean forces. For harmonic bias potential the distributions from individual windows can be approximated by Gaussian functions and the free-energy gradient along the reaction coordinate can be expressed as

$$\frac{\partial A}{\partial \xi }=\overset{N_{w}}{\underset{i=1}{\sum }}\frac{N_{i}p_{j}^{b}\left(\xi \right)}{\overset{N_{w}}{\underset{j=1}{\sum }}N_{j}p_{j}^{b}\left(\xi \right)}\left[\frac{\xi -\overset{―}{\xi_{i}^{b}\;}}{\beta {\left(\sigma_{i}^{b}\right)}^{2}}-K_{i}\left(\xi -\xi_{i0}\right)\right]$$ 8

where *N*_*i*_ is the number of samples in window *i*, $\overset{―}{\xi_{i}^{b}}$ is the mean value of the biased distribution, and $\sigma_{i}^{b}$ is standard deviation. This approach often yields smoother free-energy profiles and serves as a complementary validation of the WHAM results.

### pH-dependent QM/MM model

To extend the QM/MM description of the substitution reaction to variable pH conditions, the free-energy profiles obtained from umbrella sampling were post processed within a pH-dependent thermodynamic framework following the strategy introduced in our previous studies.^45,48,78^ In this work, the transformation is applied point by point along the WHAM reaction coordinate. The corresponding pH-dependent Gibbs–Alberty free energy at a given point *x* of the reaction coordinate is expressed as:

9 $$\Delta GA^{0}\left(x,\mathrm{pH}\right)=-RT\ln\left(K^{\prime }\right)\;\mathrm{where}\;K^{\prime }=K\cdot f\left(\mathrm{pH}\right)$$

The factor *f*(pH) combines all relevant protonation branches of the system. For the present description, this factor can be expressed as:

10 $$f\left(\mathrm{pH}\right)=\frac{10^{\{pH-pK_{a}^{\left[Au-NHC-Cys/\sec\left(COO\right)\right]}\}}+1+10^{\{pK_{a}^{\left[Au-NHC-Cys/\sec\left(NH2\right)\right]}-pH\}}}{10^{\{pH-pK_{a}^{\left[Cys/\sec\left(COO\right)\right]}\}}+1+10^{\{pK_{a}^{\left[Cys\left(S\right)/\sec\left(Se\right)\right]}-pH\}}}$$

The p*K*_a_ values used in this construction were taken from the B3LYP/DZ1-level in our previous study,^45^ which is also the only electronic-structure level at which the QM/MM pH model was evaluated in the present study. This choice ensures consistency with the reference p*K*_a_ data retaining the most conservative QM/MM description available in the present work. Since in eq 10 the explored pH range is reduced to the interval from 3 to 11, it was not necessary to retain the full protonation scheme used in our previous work.^45^ In amino acids complexes with the Au–NHC, the deprotonation of amino group is significantly stabilized, leading to a substantial decrease in the corresponding p*K*_a_. As a consequence, within the physiologically relevant pH range, it is necessary to explicitly consider also the doubly deprotonated reaction branch (2dep).

In contrast to our previous QM/MM pH-dependent model for the interactions between the Hg­(II) cation and thymine,^79^ an additional factor *ω*_*s*_(*x*, pH), accounting for the ratio of reactant and product states is considered here, further improving the quality of this model. The pH-dependent free-energy profile is finally assembled as a population-weighted sum of all the protonation branches:

11 $$\Delta G^{pH}\left(x\right)=\underset{s\in \left\{pro,dep,2dep\right\}}{\sum }\omega_{s}\left(x,\mathrm{pH}\right)\cdot \Delta GA_{s}^{0}\left(x,\mathrm{pH}\right)$$

where *ω*_*s*_(*x*, pH) are the combined reactant/product weights in the given umbrella sampling window (***x***) on the reaction coordinate and pH parameter identifies the ration (or percentage) of the individual protonation branches (pro, dep, and 2dep). In this way, the varying molar fractions of reactant- and product-like states are also taken into account.

## Results and Discussion

This work explores the reaction of a model Au­(I)–NHC complex with Cys and Sec amino acids. The substitution reaction, schematically depicted in Figure 1, is addressed in the present study, mainly using the QM/MM method, including pH-dependent model. In addition, reaction energetics were systematically compared using different combinations of DFT functional and basis sets. Particular emphasis is placed on the internal consistency and robustness of the energetic trends and, importantly, on their direct comparison with QM/MM simulations performed using identical electronic structure settings. Within this more complex model, conformational contributions to the entropy due to the explicit solvation approach are also included. Therefore, these results can be considered as an ultimate confirmation of the computational model used in our previous study.^45^

**Figure 1:**
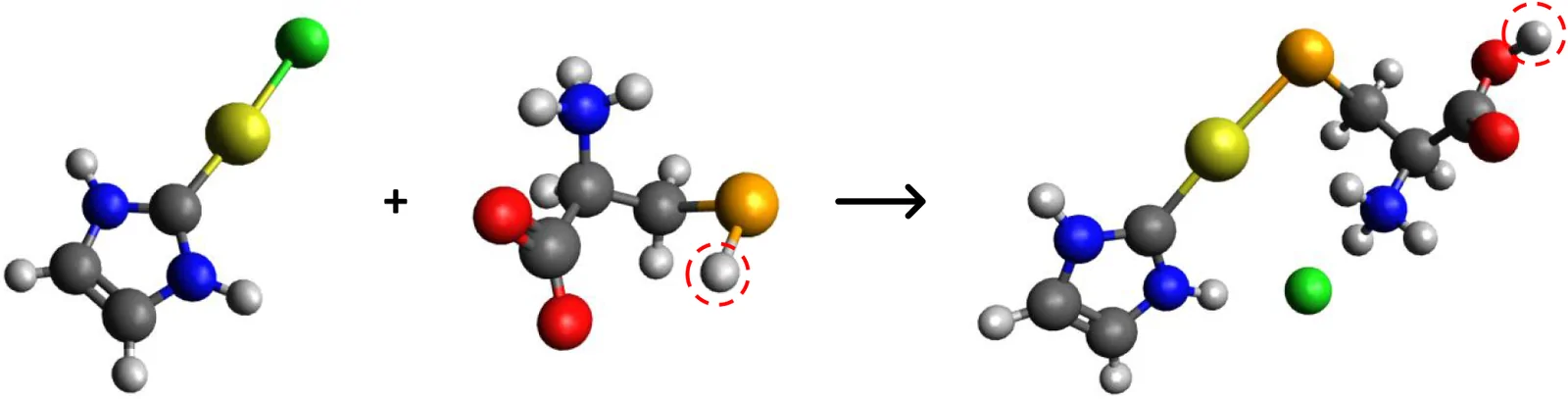
Schematic representation of the substitution reaction of the Au­(I)–NHC complex with selenocysteine in its protonated form (pro). Color coding: C (black), H (white), N (blue), Cl (green), Se/S (orange), O (red), Au (yellow).

### QM Models

The substitution reaction between the Au­(I)–NHC complex and Cys/Sec was investigated within an aqueous environment using an implicit solvation model. This setup reflects the intrinsic reaction energetics under solvated conditions and serves as the reference for subsequent comparison with QM/MM simulations.

All combinations of exchange–correlation functionals and basis sets described in the Computational Details section were systematically explored. Full details considering all explored models can be found in the Supporting Information. Here, only the most important results using the B3LYP hybrid functional in combination with the TZ, DZ1, and DZ3 basis sets (in decreasing flexibility) are presented.

The substitution reaction proceeds via a single-barrier mechanism in which the chloride ligand is displaced by the sulfur or selenium donor atom of the amino acid. For each functional–basis combination, reactant, transition state, and product supermolecular complexes were fully optimized, and their character was confirmed by vibrational analysis. The transition state is characterized by a single imaginary frequency corresponding to the antisymmetric stretching mode involving the simultaneous elongation of the Au–Cl bond together and shortening of the Au–S­(Se) bond, and vice versa. Activation free energies were determined relative to the corresponding reactant complexes, and complete reaction energy profiles were constructed for both Cys and Sec amino acids.

The activation and reaction free energies derived from Gibbs free energies, including Wertz entropy corrections, are summarized for six reaction variants considered in this part of study, namely interactions of the Au­(I)–NHC complex with pro-Cys, pro-Sec, dep-Sec, dep-Cys, 2dep-Cys and 2dep-Sec forms of amino acids in Table 1 and for all 20 functional–basis set combinations in Table S1. Closer inspection of the data collected in these tables reveals that substitution reactions involving Sec generally proceed with slightly lower activation free energies than the corresponding reactions with Cys, in all three (de)­protonated states. This preference can be rationalized in terms of fundamental hard–soft acid–base (HSAB) principle. Au­(I) behaves as a soft Lewis acid, and selenium, particularly in its anionic selenolate form, is a softer and more polarizable donor than sulfur in its thiolate form. The stronger Au–Se interaction stabilizes the developing metal-chalcogen bond along the reaction coordinate, lowering the transition-state energy relative to the corresponding Au–S pathway.

**Table 1:** Activation and Reaction Free Energies (ΔG_a_ and ΔG_r_) Including Wertz Entropy Corrections Using the B3LYP Functional with the Three Most Important Basis Set within Three Substitution Pathways (Protonated, Deprotonated and Twice Deprotonated Cys and Sec)^a^

	TZ		DZ3		DZ1	
	ΔG_a_	ΔG_r_	ΔG_a_	ΔG_r_	ΔG_a_	ΔG_r_
pro-Cys	11.4	–3.5	10.9	–3.7	11.2	–4.1
pro-Sec	11.3	–7.6	9.9	–8.1	10.0	–8.0
dep-Cys	7.4	–14.6	5.5	–16.3	6.7	–15.2
dep-Sec	6.6	–14.7	5.2	–15.8	6.3	–15.1
2dep-Cys	5.9	–12.8	5.0	–15.5	5.5	–12.4
2dep-Sec	5.2	–11.5	4.7	–13.3	4.3	–12.3

a All values are given in kcal mol-1

A second, more robust trend concerns the protonation state of the interacting amino acid. Across all selected functionals and basis sets, pro-Cys and pro-Sec consistently exhibit substantially higher activation free energies than their deprotonated counterparts. Actually, the lower the number of active protons, the lower the transition barrier is. This behavior directly reflects the enhanced nucleophilicity of the thiolate and selenolate anions compared with the neutral thiol and selenol forms. The increased electron density available for donation into the vacant coordination site at Au­(I) facilitates chloride displacement and lowers the transition-state barrier. In parallel, the reaction free energies demonstrate that substitution reactions involving deprotonated species are markedly more exergonic than those involving protonated species. Nevertheless, the second deprotonation at the amino group site does not make the reaction more exergonic than in the singly deprotonated forms.

Table S1 also reveals systematic basis-set effects. In general, a gradual decrease in computed activation barriers is observed when moving from the triple-ζ level to progressively reduced double-ζ variants and finally to LANL2DZ. The magnitude of this decrease is typically moderate; for most reaction branches, the change in activation free energy between triple-ζ and DZ3 does not exceed approximately 2 kcal·mol^–1^. Larger deviations from the triple-ζ reference are usually observed for the LANL2DZ basis set, more frequently affecting the reaction free energies than the activation barriers. The decrease of both activation and reaction free energies upon basis-set reduction is somewhat more pronounced for deprotonated species than for protonated ones. This behavior is consistent with the greater sensitivity of anionic, more diffuse electron densities to the presence or removal of diffuse basis functions, although the overall energy shifts remain limited in magnitude. Among the compared functionals, B3LYP exhibits the smallest deviations between triple-ζ and DZ3 for both activation and reaction energies, whereas B3PW91 and PBE0 show relatively larger shifts upon basis-set reduction. The triple-ζ level can therefore be regarded as a conservative reference, while the double-ζ variants, particularly DZ3, retain a high degree of consistency with the reference results.

The DFT functional dependence further modulates the quantitative results. M06–2X generally predicts less exergonic reaction free energies compared with B3LYP, B3PW91, and PBE0, and in combination with double-ζ basis sets it occasionally inverts the Sec/Cys ordering in the deprotonated branches. PBE0, in contrast, largely follows the trends observed for B3LYP and B3PW91, with only a single near-degeneracy observed at the triple-ζ level in the protonated branch. Overall, the variations between functionals remain within the typical uncertainty range of density functional theory, emphasizing that the mechanistic conclusions should be drawn from consistent trends across the full ensemble of the functional/basis-set levels rather than from a particular level.

### QM/MM Approach

In contrast to the pure QM calculations where an implicit solvent model was employed, the QM/MM simulations explicitly account for the aqueous environment and therefore require substantially higher computational effort due to statistical sampling along the reaction coordinate. For this reason, not all functional-basis set combinations explored at the QM level could also be considered in the QM/MM framework.

The triple-ζ basis set, although serving as a reliable reference in the QM study, could not be employed in QM/MM simulations due to its prohibitive computational cost in combination with molecular dynamics. QM/MM umbrella sampling requires extensive trajectory generation for each reaction window, making the triple-ζ description of the QM region computationally infeasible for the present system size and sampling length. The B3LYP and PBE0 functionals were selected for the QM/MM calculations. The M06–2X functional was not considered further in the QM/MM stage, as it exhibited less consistent Sec/Cys ordering and systematically less exergonic reaction energies at the QM level, particularly in combination with reduced double-ζ basis sets. Similarly, B3PW91 was not pursued in QM/MM simulations due to its comparatively larger energetic deviations upon reduction from triple-ζ to DZ3, indicating a higher sensitivity to basis-set contraction relative to B3LYP. The PBE0 functional, despite showing somewhat larger shifts between triple-ζ and reduced basis sets compared with B3LYP, was retained because of its computational efficiency and its overall consistency in reproducing qualitative mechanistic trends.

Inspection of the QM/MM free-energy profiles summarized in Tables 2 and S2 reveals that the effect of explicit solvation on activation barriers depends on both the evaluation method and the protonation state. At the UI level, activation barriers are systematically lower than the corresponding QM implicit-solvent values across all studied systems. In contrast, WHAM results show a more differentiated behavior: for protonated systems, the WHAM activation free energies are often comparable to or higher than the QM reference, whereas for deprotonated pathways, the barriers are consistently reduced relative to QM. The barrier reduction is therefore more pronounced for the deprotonated reaction pathways, where the anionic nucleophiles benefit from additional stabilization by the surrounding solvent environment. For deprotonated Cys and Sec, all examined QM/MM levels predict lower activation free energies than their pure QM counterparts, indicating that explicit solvent stabilization plays a significant role in facilitating chloride displacement, cf. Table S2. This effect appears to arise from additional stabilization of the anionic charge distribution in the deprotonated pathways. Importantly, the Sec < Cys kinetic preference observed at the QM level is retained within the QM/MM framework for most methodological combinations.

**Table 2:** Activation and Reaction Free Energies (ΔG_a_ and ΔG_r_) Obtained from QM/MM Calculations Using Both WHAM and UI at the B3LYP/DZ1 Level^a^

	WH (ΔG_a_)	UI (ΔG_a_)	WH (ΔG_r_)	UI (ΔG_r_)
pro-Cys	11.4	10.6	–3.1	–4.0
pro-Sec	9.7	8.7	–4.1	–5.4
dep-Cys	6.0	4.7	–17.1	–18.9
dep-Sec	4.2	3.2	–20.0	–21.4
2dep-Cys	5.0	4.0	–24.5	–26.7
2dep-Sec	4.1	3.1	–25.0	–26.7

a All values are given in kcal mol^–1^

The protonated reaction branch displays a more complex behavior. While QM/MM barriers are generally comparable to the QM reference values, larger deviations between QM and QM/MM descriptions for reaction free energies are observed for selected computational models. In particular, PBE0/LANL2DZ exhibits markedly less favorable reaction free energies for protonated pathways, occasionally predicting strongly endergonic profiles, whereas the corresponding QM results indicate exergonic behavior (see Table S2). In these cases, the discrepancy between QM and QM/MM descriptions becomes substantial. A similar, though less pronounced, tendency is observed for PBE0/DZ1, where protonated reaction free energies approach thermoneutrality in QM/MM despite the clearly exergonic character is predicted at the QM level. Notably, these inconsistencies are primarily confined to protonated pathways; deprotonated reactions remain qualitatively consistent across QM and QM/MM descriptions despite more pronounced exoergicity in the QM/MM case. Larger product stabilization is probably associated with higher conformational entropy or stronger electrostatic interactions between the solvent molecules and the more negatively charged complexes, especially in the 2dep case.

From a computational perspective, significant differences in performance are observed between the levels explored. PBE0/LANL2DZ represents the fastest combination (nevertheless with largest discrepancies for the protonated branch as mentioned above), followed closely by B3LYP/DZ3. Among the chosen approaches, B3LYP/DZ3 emerges as a particularly favorable compromise. While the reaction free energy for protonated Cys is somewhat less exergonic than predicted at the QM level, the general agreement in activation barriers and qualitative thermodynamic trends remains robust. At the same time, the computational efficiency of DZ3 makes this level substantially more practical for extended QM/MM sampling, being approximately four times faster than DZ1. This difference is already relevant for the present system, where a single umbrella-sampling window for one simulation run (20,000 steps using 32 processor cores with spec ≈ 7.0 according to methodology SPEC CPU 2017) requires approximately 2 weeks at the DZ1 level. For larger systems, however, which we ultimately aim to address in future work, the saving in computational cost will become considerably more important, as the corresponding simulations are expected to extend from weeks to months. In view of this prospective scaling, and together with the previously established consistency of DZ3 relative to the triple-ζ reference at the QM level, B3LYP/DZ3 offers the most balanced combination of accuracy, methodological stability, and computational feasibility.

In contrast, PBE0/DZ1 and B3LYP/DZ1 are substantially more demanding due to the more extended double-ζ basis sets applied to all atoms in the QM region. Although PBE0 is marginally faster than B3LYP for identical basis sets, its slightly lower reliability for protonated reaction energetics in QM/MM calculations diminishes this advantage. The B3LYP/DZ1 computational level provides good agreement with QM trends but at a considerably higher computational cost. Nevertheless, this computational level was chosen for QM/MM evaluation of the pH model below.

### pH-Dependent QM/MM Model

The main goal of this study is to determine the dependence of the free energy on the pH of the surrounding environment, which can be obtained via Legendre transformation between chemical potential and amount of substance as introduced earlier.^48,49,78^ In this way, the canonical Gibbs free energy is transformed into the grand-canonical Gibbs–Alberty free energy, where pH value is inner function of this thermodynamic potential (together with pressure and temperature). If all the necessary p*K*_a_ values are known the pH-dependent Gibbs–Alberty profiles can be constructed according to eqs 9 and 10. As mentioned above, these values were taken for the same computational approach from our previous study^45^ and resulting curves are displayed in Figure 2. This figure demonstrates a change in the thermodynamic preference between the sulfur- and selenium-containing systems or, in other words, a reaction switch based on pH value of the solution. At lower pH, the Sec branch is favored for coordination to the Au­(I) cation, whereas at higher pH the Cys coordination becomes more stable, with the crossover occurring at approximately *pH* ≈ 8. This central feature is consistent with our previous study, in which only QM methods were employed.^45^ This strongly supports the consistency of the present QM/MM treatment.

**Figure 2:**
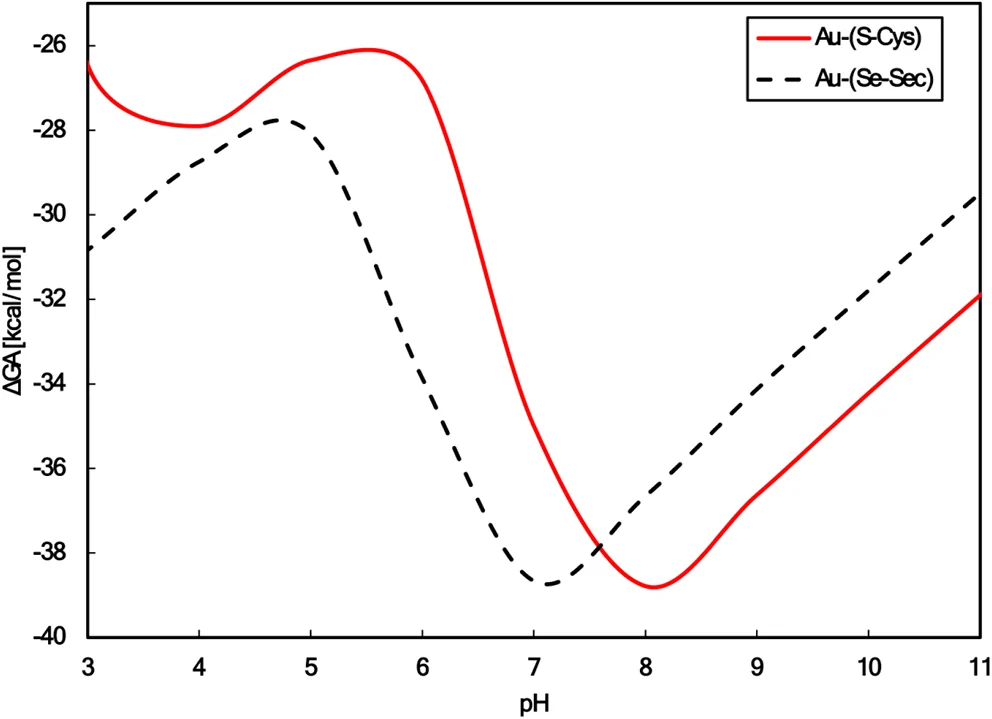
pH-dependent Gibbs–Alberty reaction free energy for the Au–S–Cys and Au–Se–Sec systems obtained from the QM/MM pH-dependent model at the B3LYP/DZ1 level. The curves were constructed from the WHAM free-energy profiles The profiles are shown in the pH range 3–11, where the TrxR enzyme is expecting to operate.

Unlike the monotonic QM curves reported earlier,^45^ the present profiles exhibit a more structured pH dependence, with local changes in slope reflecting the explicit-solvent free-energy surface and the reduced protonation manifold used here. In this sense, the QM/MM results preserve the key qualitative conclusion of the above-mentioned study^45^ while showing that the detailed pH response is more sensitive to the underlying free-energy landscape than the stationary-point QM picture alone would suggest.

## Conclusions

In this work, the substitution reaction of the Au­(I)–NHC complex with cysteine and selenocysteine was investigated at the QM and QM/MM levels. The main goal of this study was to construct a pH-dependent model based on QM/MM free-energy profiles. For this purpose, several steps were introduced. At the QM level, two dominant trends clearly emerged across the investigated functional–basis set combinations. The substitution reactions involving Sec generally proceed with lower activation free energies than the corresponding Cys systems in both protonated and deprotonated forms. This Sec < Cys preference is consistently observed at the triple-ζ level but becomes partially method-dependent at reduced basis-set levels.

The second important trend is the strong effect of protonation state. Deprotonated Cys and Sec systematically exhibit significantly lower activation barriers than their protonated counterparts, accompanied by markedly more exergonic reaction free energies.

Systematic basis-set effects are also evident. A gradual decrease in activation free energies is observed when moving from triple-ζ to reduced double-ζ descriptions and further to LANL2DZ, with the largest deviations appearing for the latter. This concerns also the reaction energies. These effects are more pronounced for deprotonated species. Among the considered functionals, B3LYP shows the smallest sensitivity to basis-set reduction, whereas B3PW91 and PBE0 exhibit larger shifts. The M06–2X functional predicts systematically less exergonic reaction free energies and, in combination with smaller basis sets, partially disrupts the Sec/Cys preference ordering.

At the QM/MM level, the effect of explicit solvation is introduced demonstrating the importance of conformational entropy contributions. The effect of solvation on the activation barriers depends on both the evaluation method and the protonation state. At the UI level, activation barriers are consistently lower than the corresponding QM values for all studied systems. In contrast, WHAM results show a more differentiated behavior; for deprotonated pathways, the barriers are systematically reduced relative to QM, whereas for protonated systems they are often comparable to or slightly higher than the QM reference. Despite these differences, the Sec < Cys kinetic preference observed at the QM level is largely preserved within the QM/MM framework. In contrast, the reaction free energies of the protonated pathways exhibit a more method-dependent behavior. While activation barriers remain comparable to QM values, significant deviations are visible in reaction free energies for selected methodological combinations. Here, a partial dependence on the total charge of the amino acid can be observed, in contrast to the pure QM results. This can be linked with explicit solvation model where higher conformational entropy contributes to the higher product stability caused by a more convenient orientational arrangement of dipole moments of surrounding water molecules around Au­(I) complex with coordinated (negatively charged) amino acid.

These results allow a clear identification of an optimal QM/MM strategy. The comparison of selected QM/MM computational levels shows that the B3LYP/DZ1 model provides good agreement with QM trends but at a substantially higher computational cost. In this context, B3LYP/DZ3 emerges as the most balanced compromise, maintaining consistent activation barriers and qualitative thermodynamic trends while significantly reducing computational demands. This model appears to be suitable for larger systems from the perspective of activity of the whole TrxR enzyme. Nevertheless, the pH-dependent model was evaluated at the B3LYP/DZ1 level, since this more demanding approach still remains computationally feasible for the present relatively small system and provides the most accurate QM/MM description used in this work.

The pH-dependent model constructed on the basis of QM/MM free-energy profiles reveals a clear exchange of thermodynamic preference between the selenium- and sulfur-containing systems. At lower pH, the Sec pathway is favored, whereas at higher pH the Cys pathway becomes more stable, with the crossover occurring at pH ≈ 8. This behavior is in good agreement with our previous study, in which pure QM methods were employed, confirming that the key pH-dependent trend is also preserved within the explicit-solvent QM/MM framework. In this way, the pH-controlling mechanism can be used as a switch for changing the reaction preference.

## Supplementary Material



## Notes

Additional computational details; activation and reaction free energies for all functional–basis set combinations; comparison of QM/MM and QM results; QM/MM free-energy profiles for selected computational models (PDF)
