# Supplementary material for: Reaction of Cysteine and Selenocysteine with a Model Au(I)-N-Heterocyclic Carbene Complex: A QM and QM/MM MD Comparative Study
Source: Inorg Chem. 2026 Oct 15;65(42):25080–9. doi: 10.1021/acs.inorgchem.6c04543 (PMC13715579; doi:10.1021/acs.inorgchem.6c04543)

Supporting Information

Reaction of Cysteine and Selenocysteine with a Model Au(I)-N-Heterocyclic Carbene Complex: A QM and QM/MM MD Comparative Study

Milan Říha[a], Markéta Munzarová[a], and Jaroslav V. Burda[b*]

[a]*Department of Chemistry, Faculty of Natural Sciences, Masaryk University, Kamenice 5, 62500 Brno, Czech Republic*
[b]*Department of Chemical Physics and Optics, Faculty of Mathematics and Physics. Charles University, Ke Karlovu 3. 121 16 Prague 2, Czech Republic*

*corresponding author: jaroslav.burda@matfyz.cuni.cz

Content of Supporting Information:

Four hybrid functionals — B3LYP, B3PW91, M06-2X, and PBE0 — were combined with five basis set schemes of increasing flexibility and varying treatment of diffuse functions. The triple-zeta reference level (**TZ**) employs 6-311++G(2df,2pd) for H, C, N, and O atoms together with corresponding Stuttgart–Dresden relativistic effective core potential for Au, Cl, and S/Se, with consistent extension of polarization and diffuse functions. The double-zeta variants include: (i) **DZ1**, based on 6-31++G(d,p) for H, C, N, and O combined with the corresponding Stuttgart–Dresden basis for Au, Cl, and S/Se also consistently extended; (ii) **DZ2**, in which diffuse functions were removed from hydrogen, carbon, nitrogen, and gold – i.e., 6-31G(d,p) was employed for H, C, and N, a corresponding Stuttgart–Dresden basis was used for Au also without diffuse functions. Only oxygen, Cl, and S/Se retained the diffuse function from (i) description; and (iii) **DZ3**, where diffuse functions were removed from all atoms except oxygen, meaning that 6-31G(d,p) was used for H, C, and N, and diffuse-free Stuttgart–Dresden basis sets were applied to Au, Cl, and S/Se. The standard LANL2DZ effective core potential basis set was additionally considered as the computationally least demanding option.

Table S1. Activation and reaction free energies (ΔG_a and ΔG_r) including Wertz entropy corrections for all 20 functional–basis set combinations and all substitution pathways (protonated, singly deprotonated, and doubly deprotonated Cys and Sec). All values are given in kcal mol^{-1}.

		TZ		DZ1		DZ2		DZ3		LANL2DZ	
		ΔG_a	ΔG_r	ΔG_a	ΔG_r	ΔG_a	ΔG_r	ΔG_a	ΔG_r	ΔG_a	ΔG_r
pro-Cys	B3LYP	11.4	-3.5	11.2	-4.1	11.2	-3.5	10.9	-3.7	11.7	-5.0
	B3PW91	11.1	-4.6	10.2	-5.7	10.3	-5.0	9.6	-5.5	10.6	-6.9
	M06-2X	11.7	-3.9	10.9	-5.0	10.9	-4.9	10.1	-5.6	10.7	-5.5
	PBE0	11.7	-5.2	11.5	-5.8	11.3	-5.7	10.6	-6.3	11.7	-7.3
pro-Sec	B3LYP	11.3	-7.6	10.0	-8.0	10.0	-7.8	9.9	-8.1	9.8	-7.8
	B3PW91	10.2	-9.1	8.8	-10.0	9.0	-9.4	8.8	-9.7	8.3	-10.0
	M06-2X	11.3	-7.8	10.2	-8.7	10.2	-8.6	10.0	-8.9	9.0	-8.2
	PBE0	11.8	-9.1	10.4	-9.9	10.3	-9.7	10.3	-9.9	9.3	-10.6
dep-Cys	B3LYP	7.4	-14.6	6.7	-15.2	6.4	-15.3	5.5	-16.3	5.3	-11.3
	B3PW91	6.8	-14.6	6.1	-15.2	5.8	-15.0	4.5	-16.1	4.1	-12.3
	M06-2X	6.6	-11.6	5.0	-11.6	5.2	-11.8	4.9	-13.3	3.7	-8.0
	PBE0	7.2	-14.5	6.5	-15.0	6.2	-14.6	5.1	-16.3	4.8	-11.6
dep-Sec	B3LYP	6.6	-14.7	6.3	-15.1	5.4	-15.1	5.2	-15.8	4.7	-12.7
	B3PW91	5.6	-14.4	5.5	-15.2	4.2	-15.1	4.2	-16.0	3.7	-13.4
	M06-2X	6.1	-11.3	5.5	-11.8	5.6	-11.2	5.3	-11.7	3.8	-9.0
	PBE0	6.6	-13.8	5.1	-15.2	5.6	-14.7	4.9	-15.4	3.9	-12.9
2dep-Cys	B3LYP	5.9	-12.8	5.5	-12.4	5.6	-14.1	5.0	-15.5	3.4	-9.0
	B3PW91	4.9	-13.1	4.7	-11.9	5.3	-13.8	2.6	-16.5	3.1	-10.4
	M06-2X	5.0	-8.5	3.8	-9.0	4.2	-10.0	2.3	-12.1	2.2	-5.7
	PBE0	5.4	-12.8	5.0	-13.8	5.0	-14.0	3.9	-15.5	3.4	-9.2
2dep-Sec	B3LYP	5.2	-11.5	4.3	-12.3	5.0	-12.8	4.7	-13.3	3.0	-9.3
	B3PW91	3.8	-11.5	2.6	-12.9	3.8	-13.4	3.2	-14.6	3.0	-10.0
	M06-2X	4.9	-7.3	4.0	-8.0	4.2	-8.7	3.5	-9.6	1.7	-6.4
	PBE0	4.6	-10.6	4.1	-11.7	3.8	-12.5	4.5	-12.8	3.0	-9.4

Table S2. Activation and reaction free energies (ΔG_a and ΔG_r) obtained from QM/MM (both WHAM and UI) calculations compared to the QM energies at the same level. All values are given in kcal mol^{-1}.

		WH	UI	WH	UI	QM ref.	
		ΔG_a	ΔG_a	ΔG_r	ΔG_r	ΔG_a	ΔG_r
pro-Cys	B3LYP/DZ3	10.9	10.2	-0.1	-1.2	10.9	-3.7
	B3LYP/DZ1	11.4	10.6	-3.1	-4.0	11.2	-4.1
	PBE0/DZ1	12.2	11.1	1.1	-0.5	11.5	-5.8
	PBE0/LANL2DZ	14.2	13.4	7.5	6.5	11.7	-7.3
pro-Sec	B3LYP/DZ3	8.2	7.4	-4.1	-5.4	9.9	-8.1
	B3LYP/DZ1	9.7	8.7	-4.1	-5.4	10.0	-8.0
	PBE0/DZ1	11.0	9.8	0.1	-1.4	10.4	-9.9
	PBE0/LANL2DZ	10.2	9.3	2.0	0.8	9.3	-10.6
dep-Cys	B3LYP/DZ3	4.6	3.8	-19.4	-20.9	5.5	-16.3
	B3LYP/DZ1	6.0	4.7	-17.1	-18.9	6.7	-15.2
	PBE0/DZ1	5.8	5.1	-18.6	-20.0	6.5	-15.0
	PBE0/LANL2DZ	3.3	2.6	-13.0	-14.2	4.8	-11.6
dep-Sec	B3LYP/DZ3	3.3	2.4	-22.1	-23.0	5.2	-15.8
	B3LYP/DZ1	4.2	3.2	-20.0	-21.4	6.3	-15.1
	PBE0/DZ1	5.0	4.3	-20.6	-21.9	5.1	-15.2
	PBE0/LANL2DZ	2.3	1.9	-18.3	-19.5	3.9	-12.9
2dep-Cys	B3LYP/DZ1	5.0	4.0	-24.5	-26.7	5.5	-12.4
2dep-Sec	B3LYP/DZ1	4.1	3.1	-25.0	-26.7	4.3	-12.3

Figure S1. Comparison of energy profiles for selected computational models:

a) QM/MM reaction profiles at the B3LYP/DZ1 level including the doubly deprotonated (2dep) systems, which were calculated only at this level.

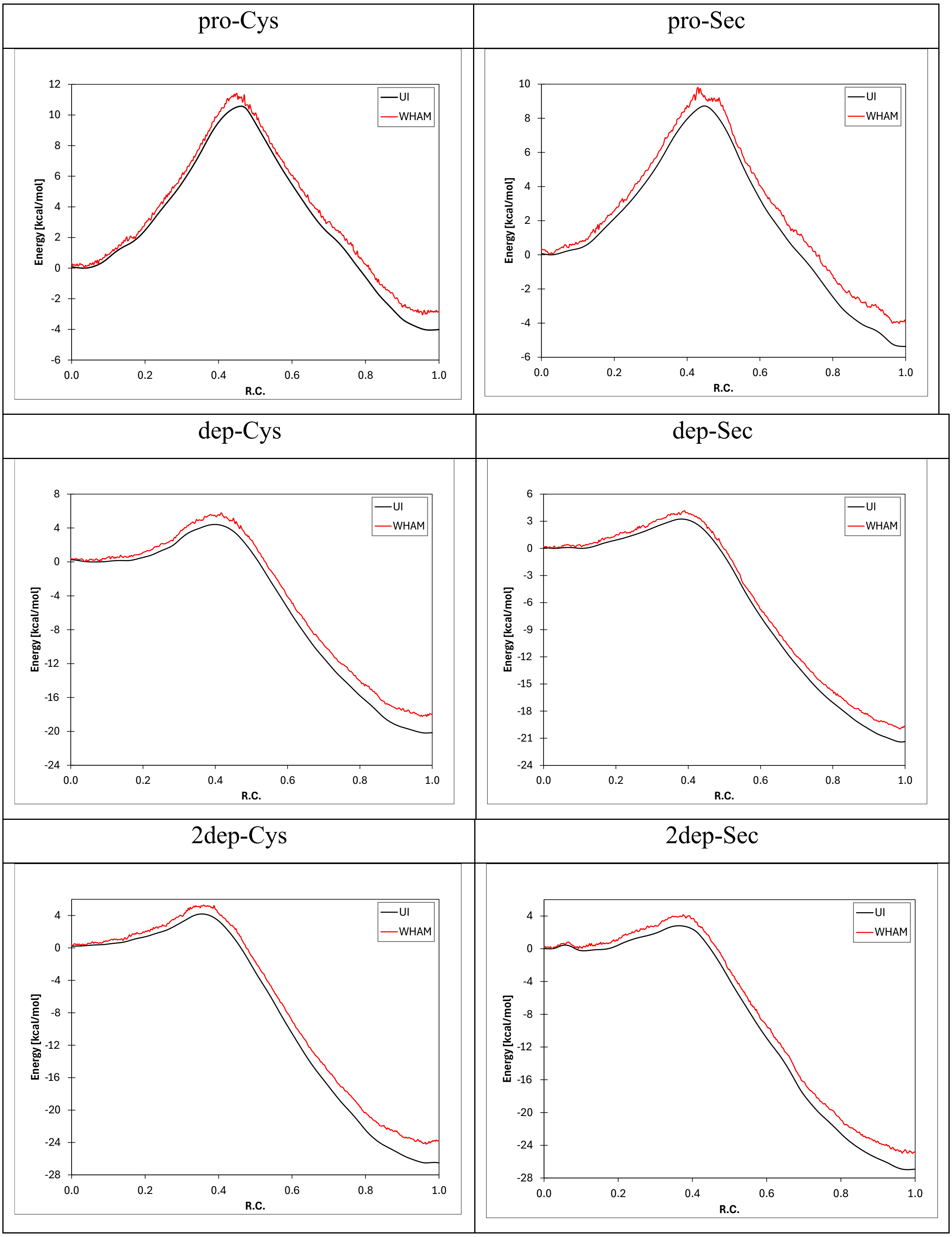

b) QM/MM reaction profiles at the B3LYP/DZ3 level.

pro-Cys

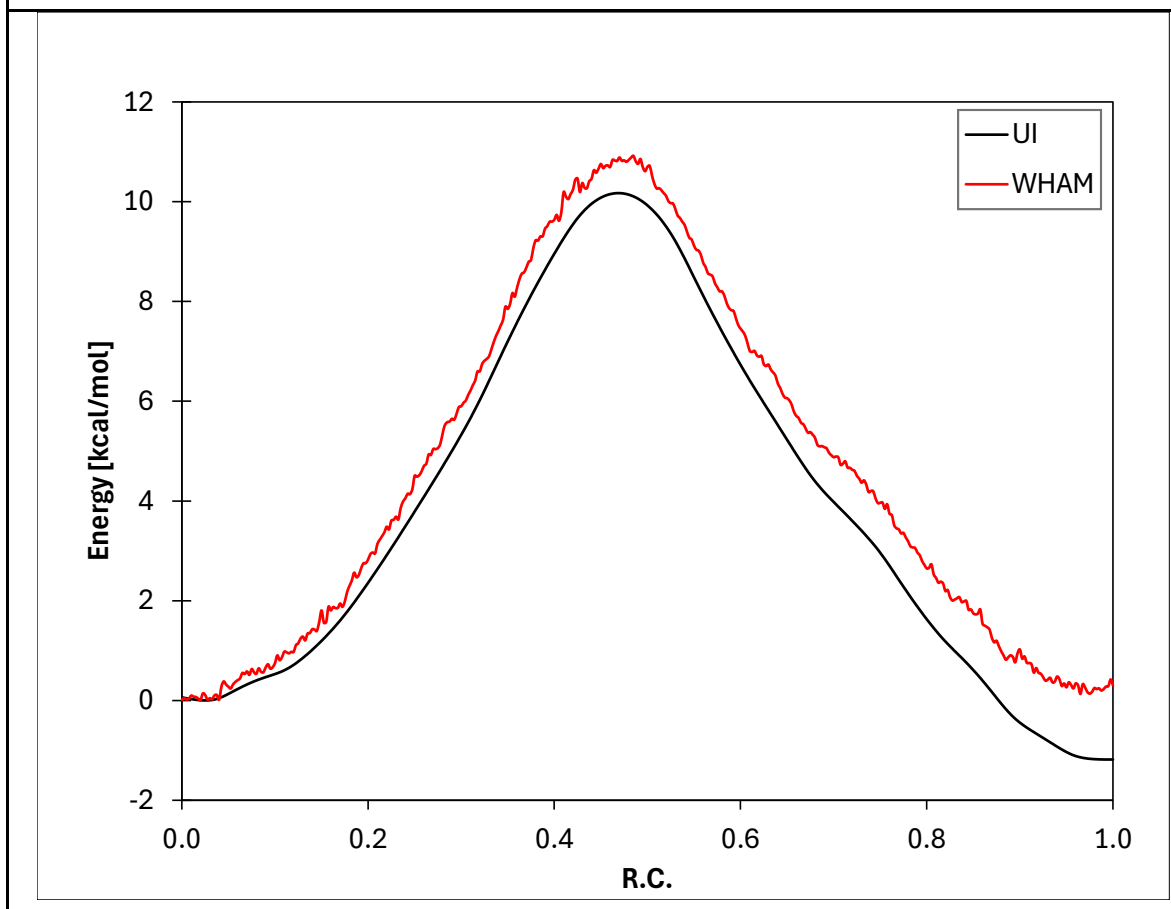

pro-Sec

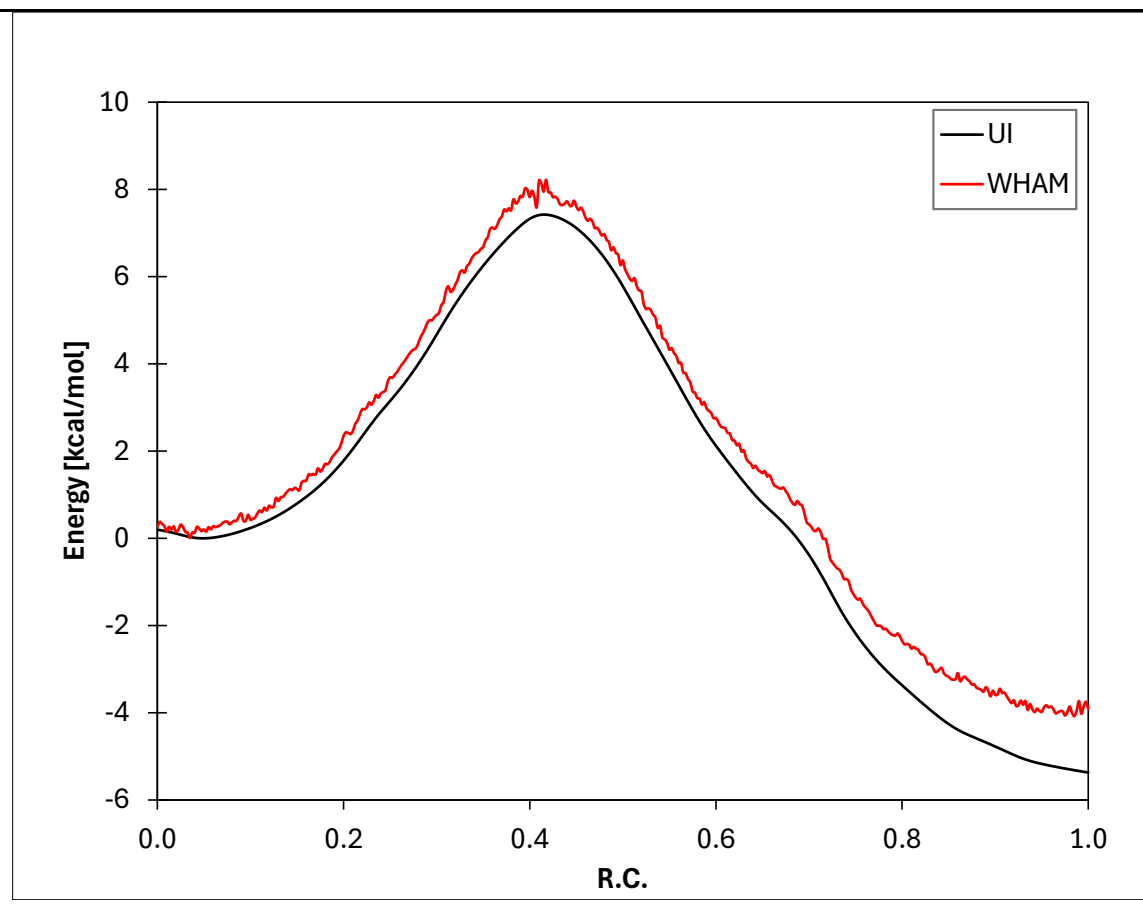

dep-Cys

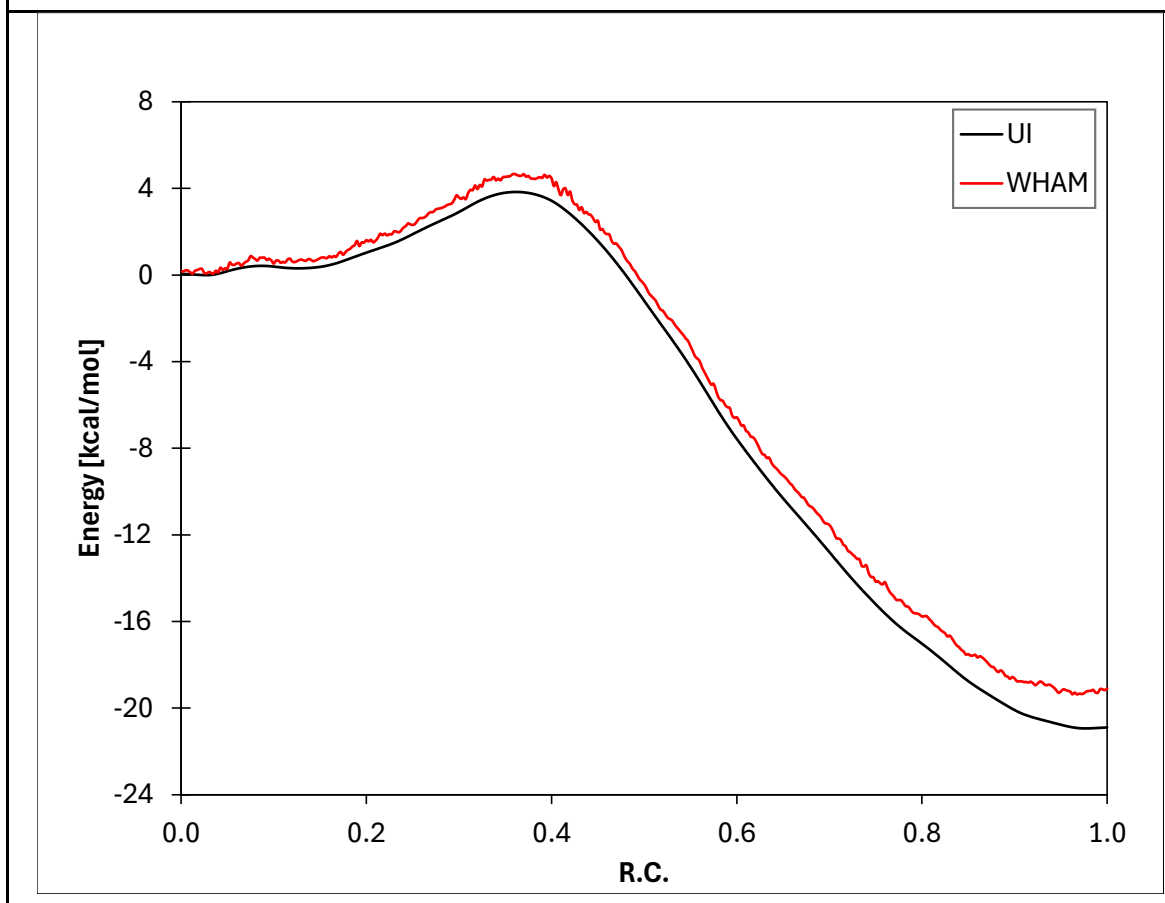

dep-Sec

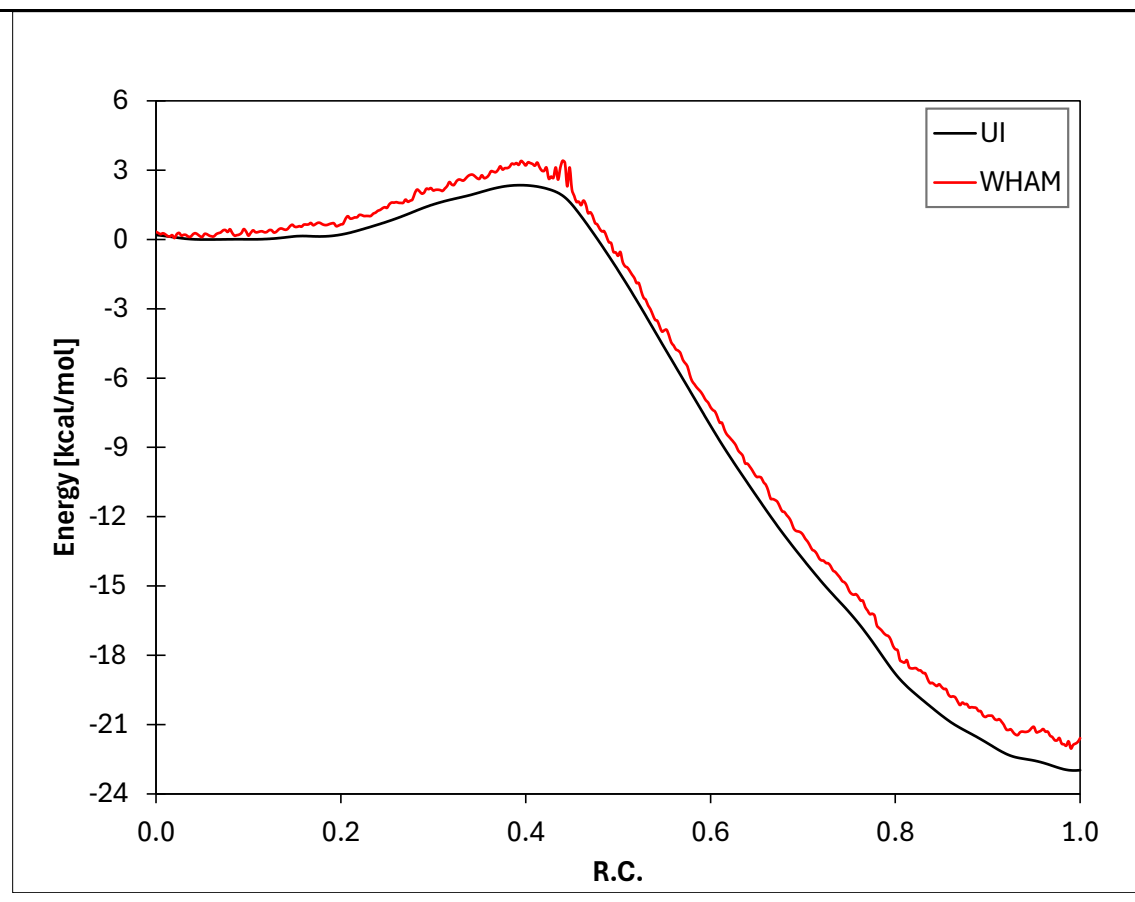

c) QM/MM reaction profiles at PBE0/DZ1 level.

pro-Cys

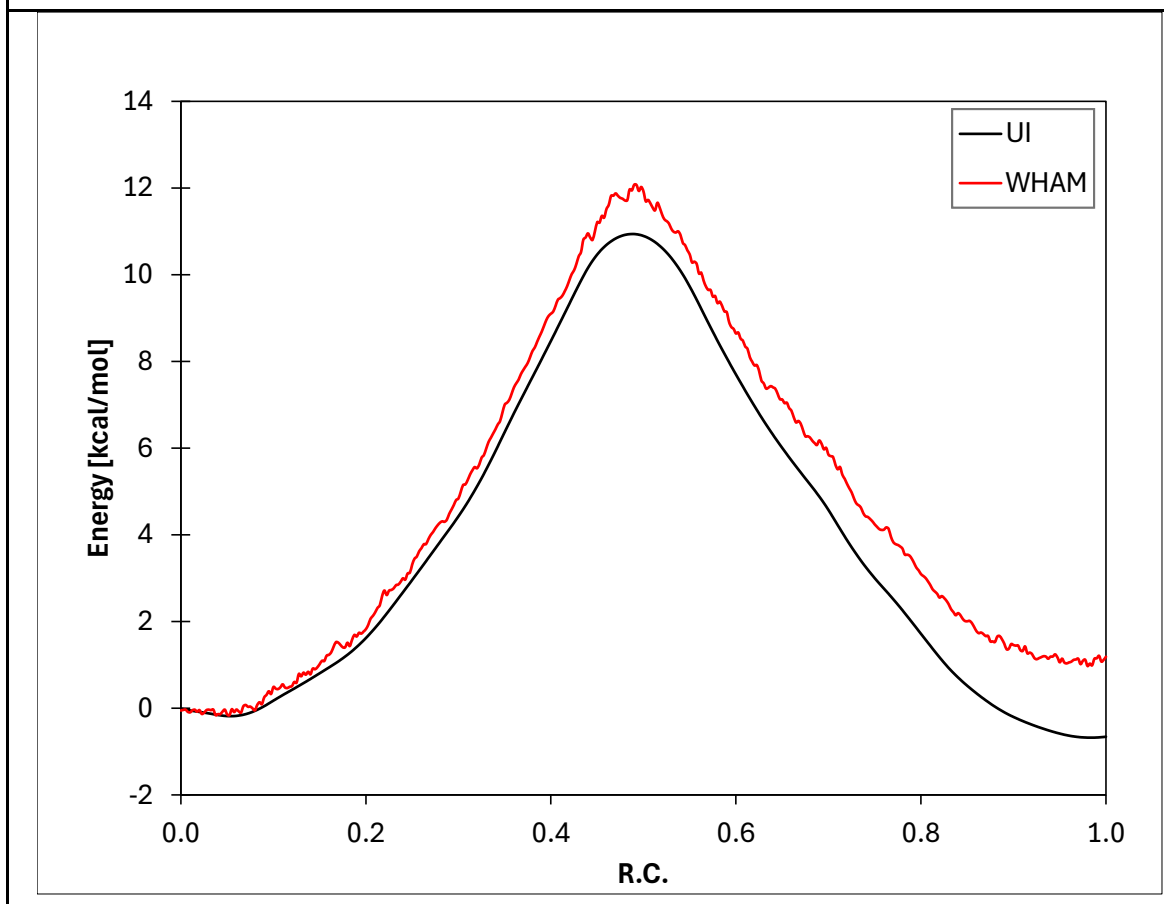

pro-Sec

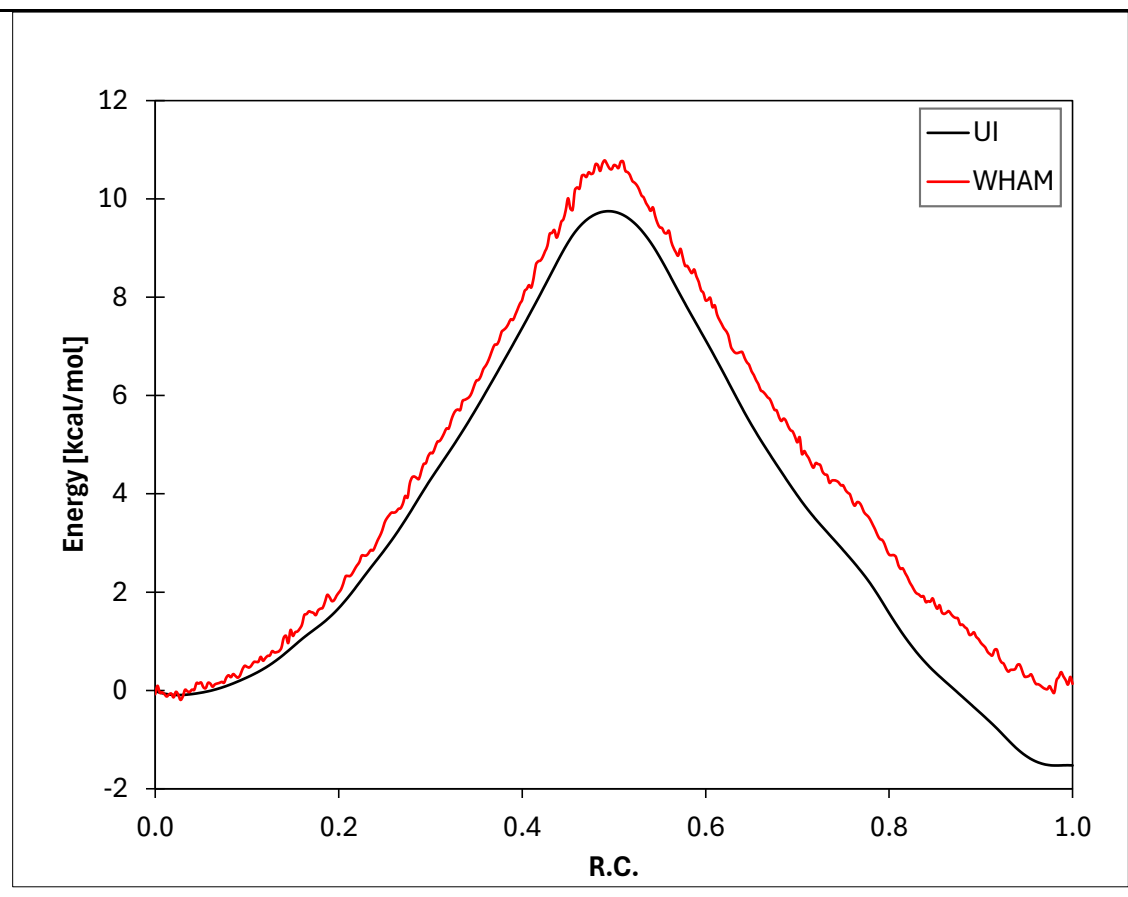

dep-Cys

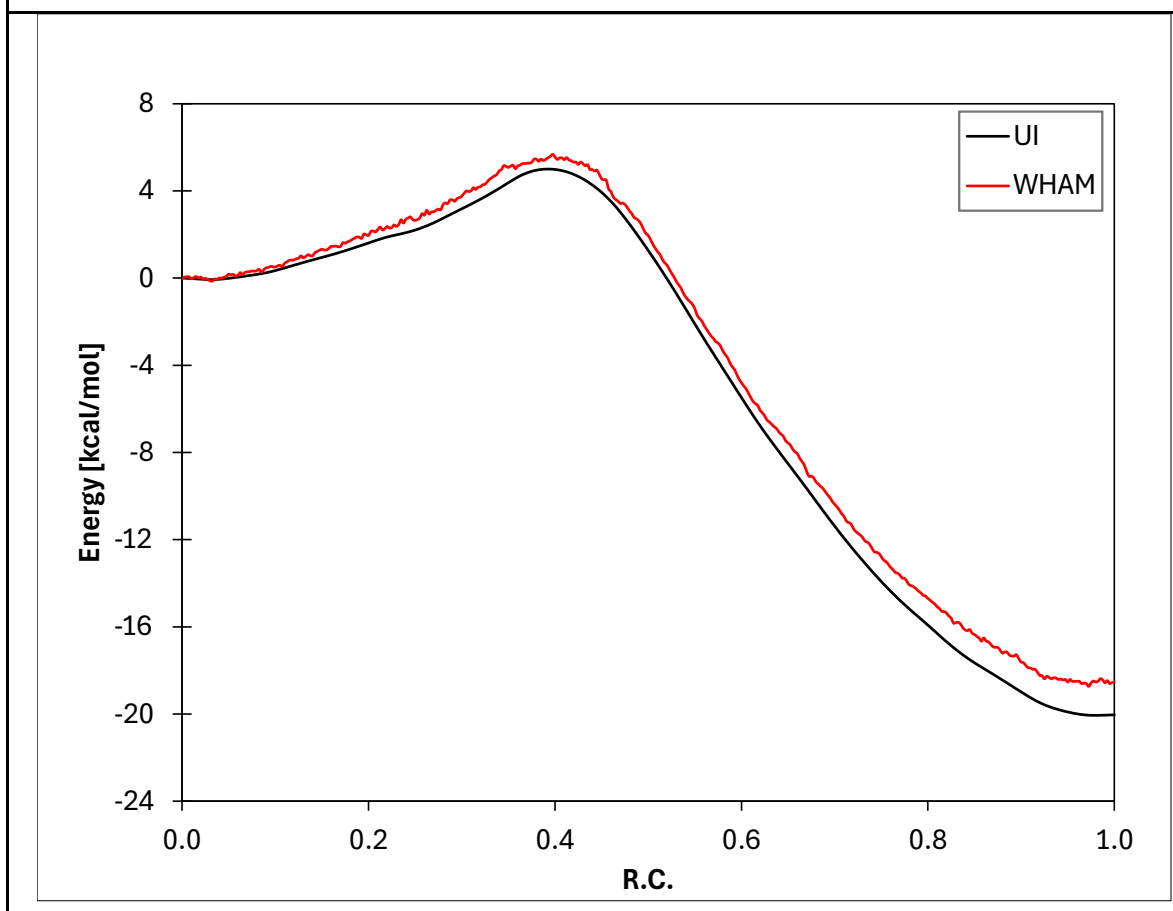

dep-Sec

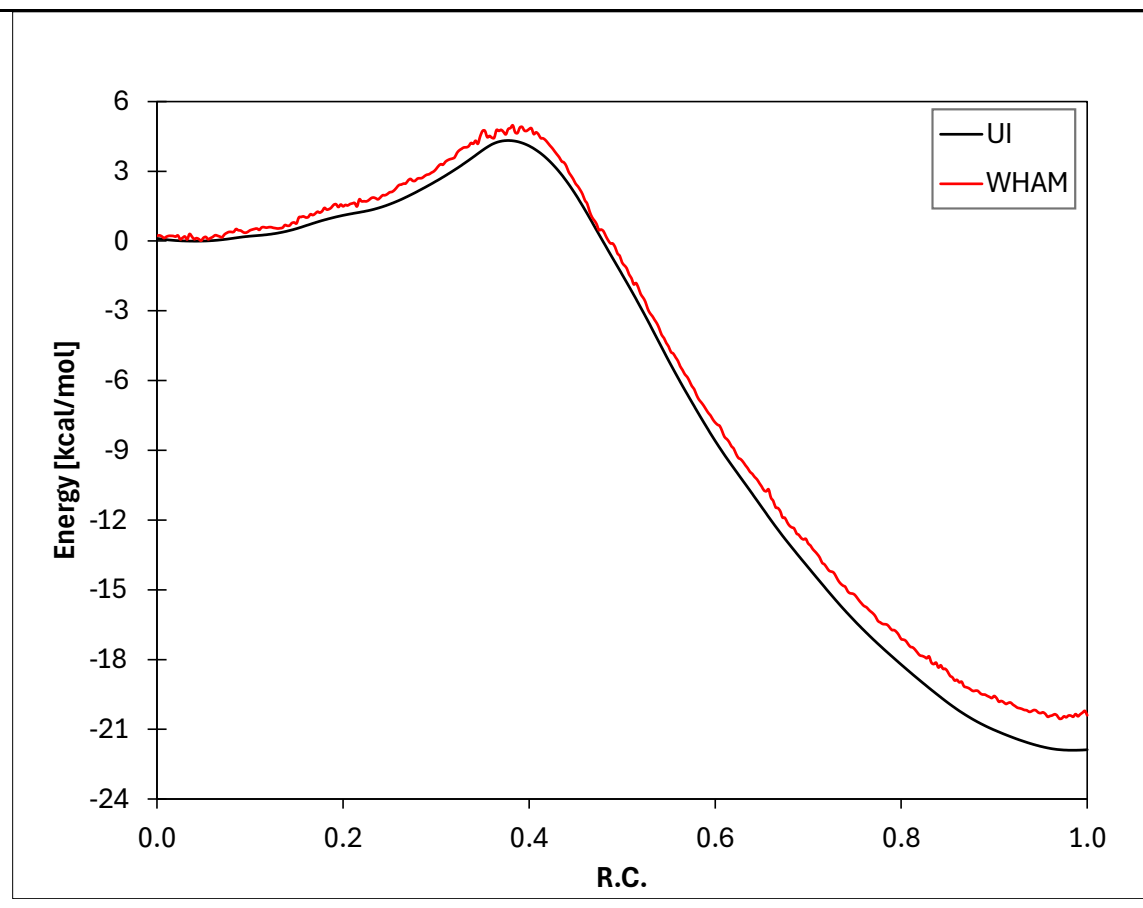

d) QM/MM reaction profiles at the PBE0/LANL2DZ level.

pro-Cys

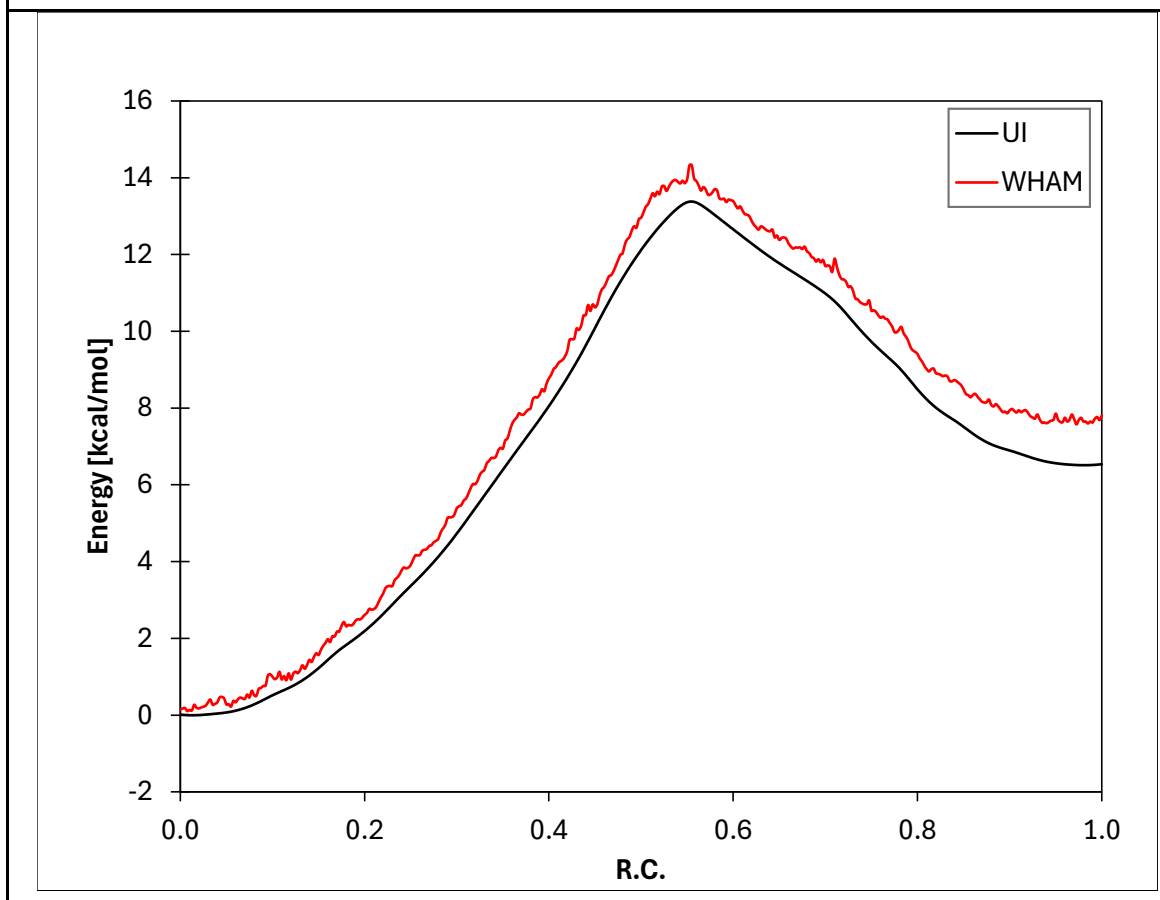

pro-Sec

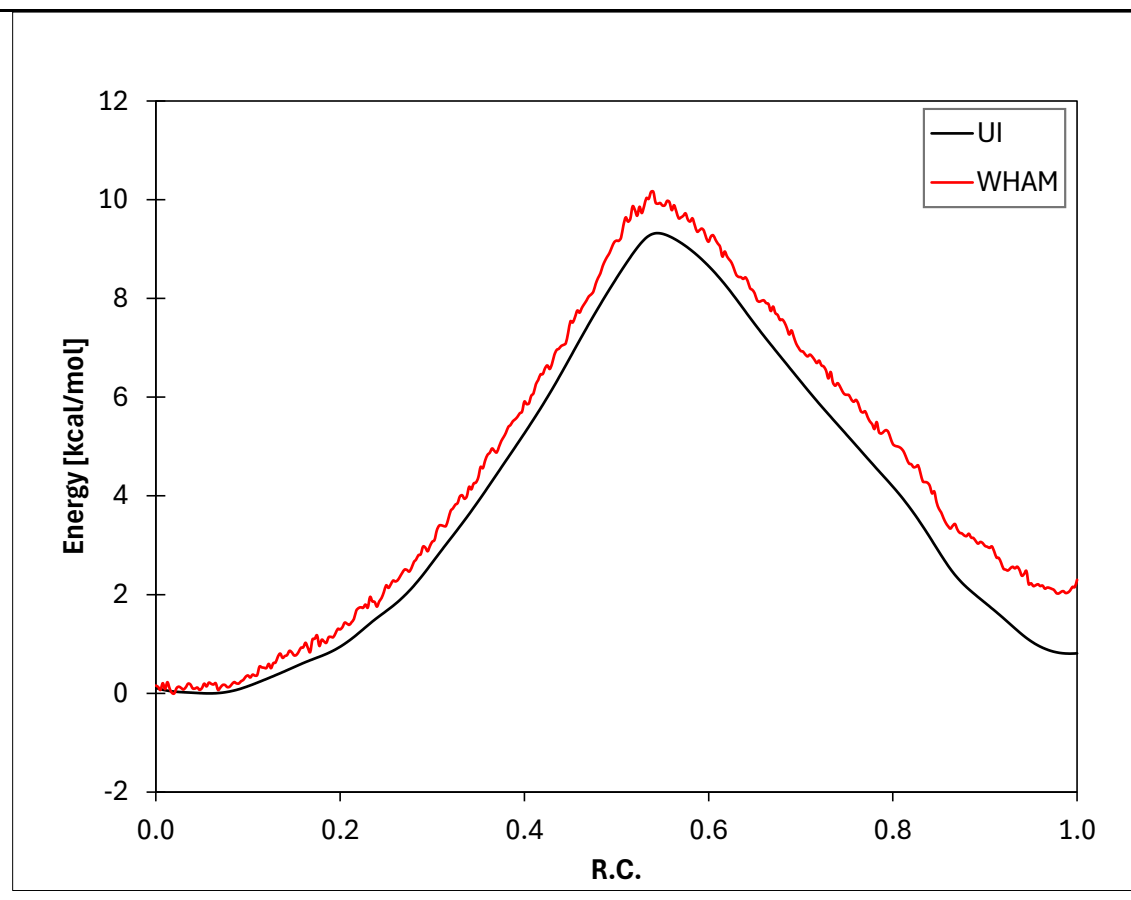

dep-Cys

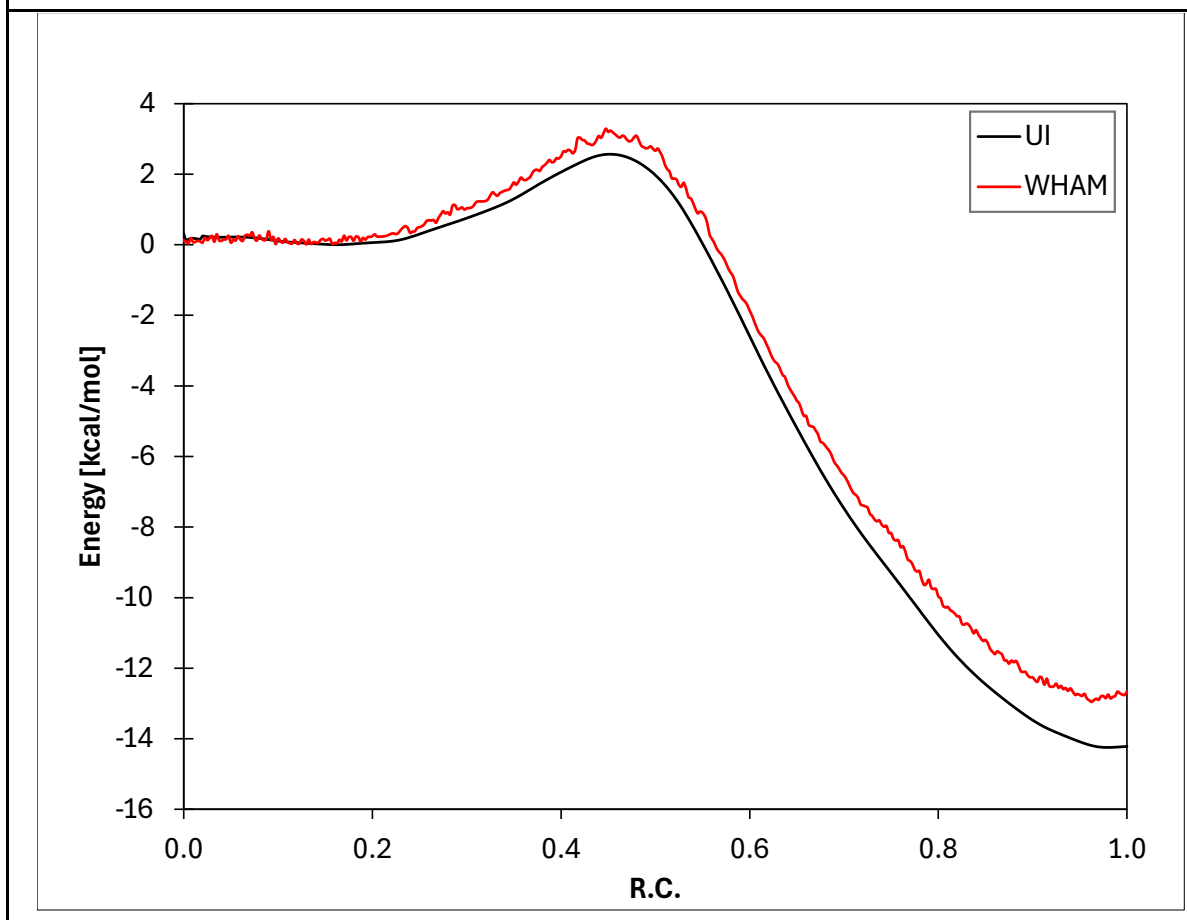

dep-Sec

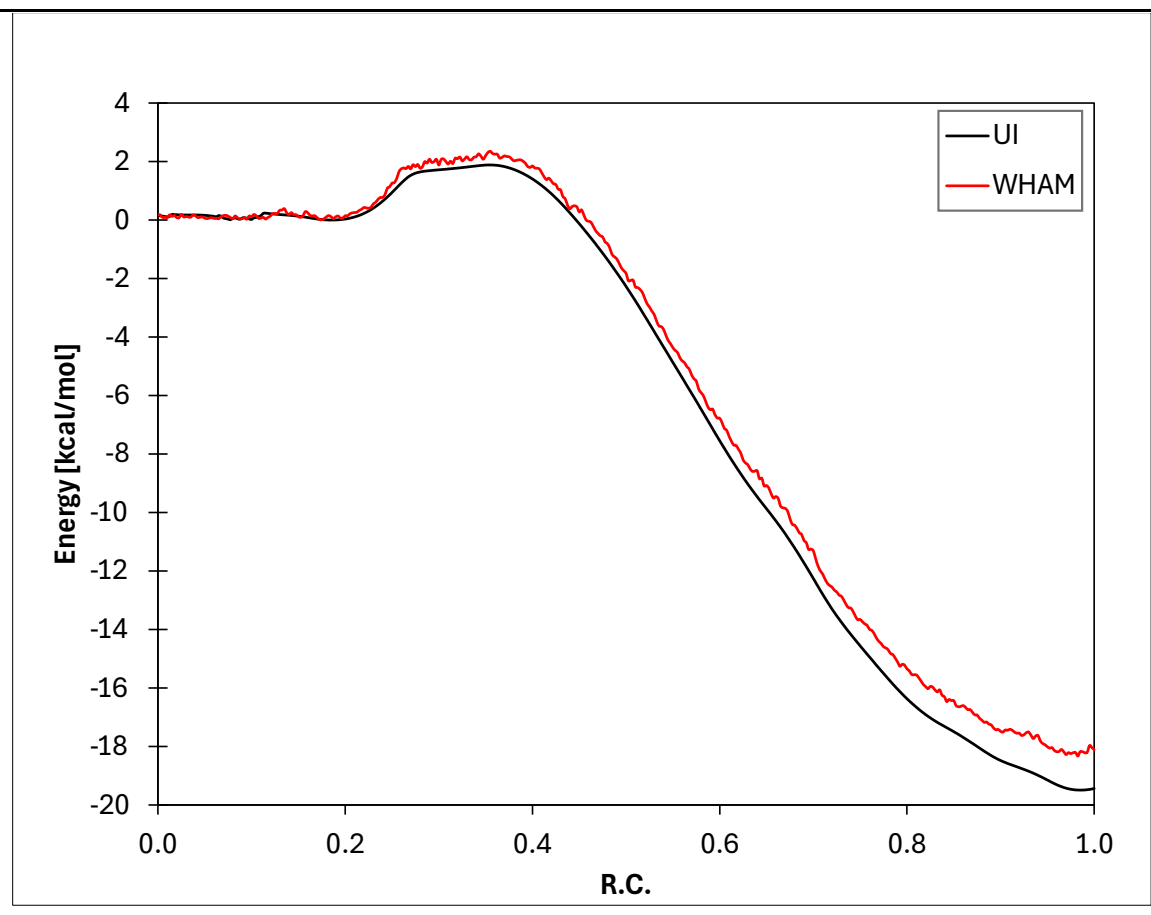

Supplement: Supplementary file 1 [file ic6c04543_si_001.pdf]
